# *MALAT1* promotes osteosarcoma development by targeting *TGFA* via *MIR376A*

**DOI:** 10.18632/oncotarget.10752

**Published:** 2016-07-21

**Authors:** Wei Luo, Hongbo He, Wenfeng Xiao, Qing Liu, Zhenhan Deng, Yaojuan Lu, Qian Wang, Qiping Zheng, Yusheng Li

**Affiliations:** ^1^ Department of Orthopedics, Xiangya Hospital, Central South University, Changsha, 410008, China; ^2^ Department of Hematological Laboratory Science, Jiangsu Key Laboratory of Medical Science and Laboratory Medicine, School of Medicine, Jiangsu University, Zhenjiang 212013, China

**Keywords:** IncRNA, MALAT1, MIR376A, osteosarcoma, TGFA

## Abstract

Metastasis-associated lung adenocarcinoma transcript 1 (*MALAT1*) is a long non-coding RNA (lncRNA) that contributes to the initiation and development of many solid tumors, including osteosarcoma (OS). Here, we showed that *MALAT1* was increased in human OS cell lines and tissues and promoted OS cell growth, while *MALAT1* knockdown suppressed OS cell growth. We also detected downregulation of *MIR376A*, a suppressor of OS growth, and upregulation of *TGFA*, a promoter of OS growth, in OS tissues. *TGFA* expression was positively correlated with *MALAT1* expression, and both were negatively correlated with *MIR376A* expression. There was a direct interaction between *MIR376A* and *MALAT1* via a putative *MIR376A* binding site within the *MALAT1* 3′-untranslated region (3′-UTR). There was also a direct interaction between *MIR376A* and the *TGFA* 3′-UTR. Thus, *MALAT1* may promote OS cell growth through inhibition of *MIR376A*, leading to increased expression of *TGFA*. Our results suggest a *MALAT1/MIR376A/TGFA* axis mediates OS cell proliferation and tumor progression.

## INTRODUCTION

More than 90% of the human DNA sequence is actively transcribed but only 2% of it encodes protein. The majority of transcripts are referred to as non-coding RNAs (ncRNAs) [1, 2]. Small non-coding RNAs, especially, microRNAs, have been studied extensively and their roles in gene regulation and cellular function have been elucidated in numerous cancers [2]. Recently, long non-coding RNAs (lncRNAs) have been reported to play important roles during development and in diseases, including cancer [3, 4]. LncRNAs can be oncogenic or function as tumor suppressors [5, 6]. Several lncRNAs play an oncogenic role in breast, gastric, colorectal, and cervical cancers [7], while others function as tumor suppressors in hepatocellular carcinoma and gastric cancer [8, 9]. In osteosarcoma (OS), expression of several lncRNAs may be upregulated [10, 11].

Recently, the lncRNA, *MALAT1* was reported to be upregulated in lung, breast, pancreas, liver, colon, gastric, uterus, cervix and prostate cancers [12, 13]. *MALAT1* may also serve as an independent prognostic biomarker for survival of these cancers. *MALAT1* expression is associated with OS cell fate, as *MALAT1* knockdown delays tumor growth in an OS xenograft model, suggesting its oncogenic role and potential as a therapeutic target [14]. *MALAT1* also promotes OS cell growth and metastasis, possibly via activation of the PI3K/AKT signaling pathway [15]. While these findings demonstrate a clear correlation between *MALAT1* and OS, the specific effect of *MALAT1* on OS tumorigenesis and the mechanisms involved remain to be determined.

In this study, we measured expression of *MALAT1*, *MIR376A* and *TGFA* in OS cell lines and tissues. We found a negative correlation between *MIR376A* and *MALAT1* or *TGFA* expression. To understand the mechanisms of *MALAT1* in the OS tumorigenesis, we detected a direct interaction between *MIR376A* and both *MALAT1* and *TGFA*, suggesting a novel mechanism of *MALAT1*, *MIR376A*, and *TGFA* in the regulation of OS cell growth.

## RESULTS

### Upregulation of *MALAT1* expression in human osteosarcoma tissues and cells

**Figure 1:**
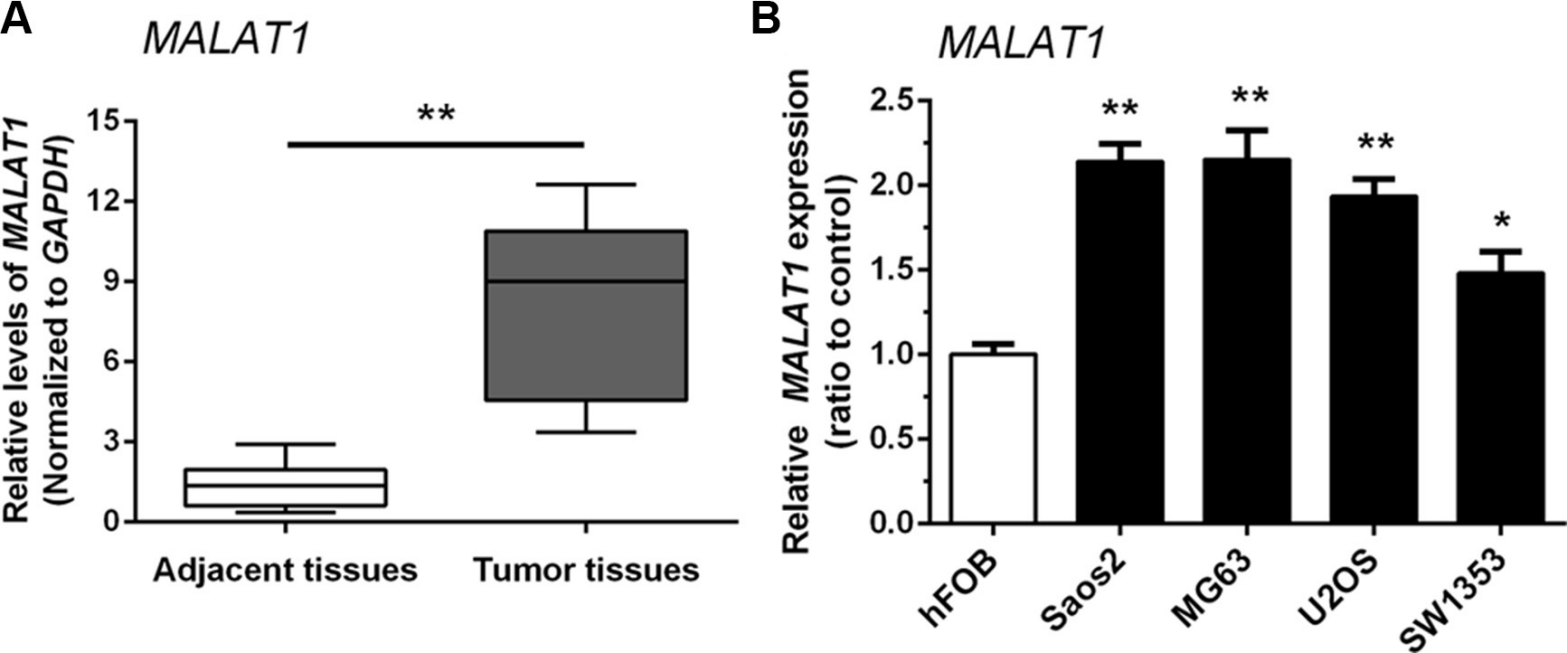
Upregulation of *MALAT1* expression in human osteosarcoma tissues and cells (**A**) *MALAT1* expression was higher in tumor tissues compared with adjacent non-tumor tissues. (**B**) *MALAT1* expression in four human OS cell lines: Saos2, MG63, SW1353 and U2OS, was upregulated compared to normal human osteoblast (hFOB) cells. Data are presented as mean ± SD of three independent experiments.

The expression of *MALAT1* in 38 paired samples (OS specimens and corresponding adjacent non-tumor tissues) was examined by real-time qPCR. *MALAT1* expression was higher in tumor tissues compared with adjacent non-tumor tissues (Figure 1A). We also examined *MALAT1* expression in four human OS cell lines: Saos2, MG63, SW1353, U2OS, and compared them with normal human osteoblast (hFOB) cells. As shown in Figure 1B, the expression of *MALAT1* was higher in all four human OS cell lines than in hFOB cells.

### *MALAT1* promotion of osteosarcoma cell growth *in vitro*

**Figure 2:**
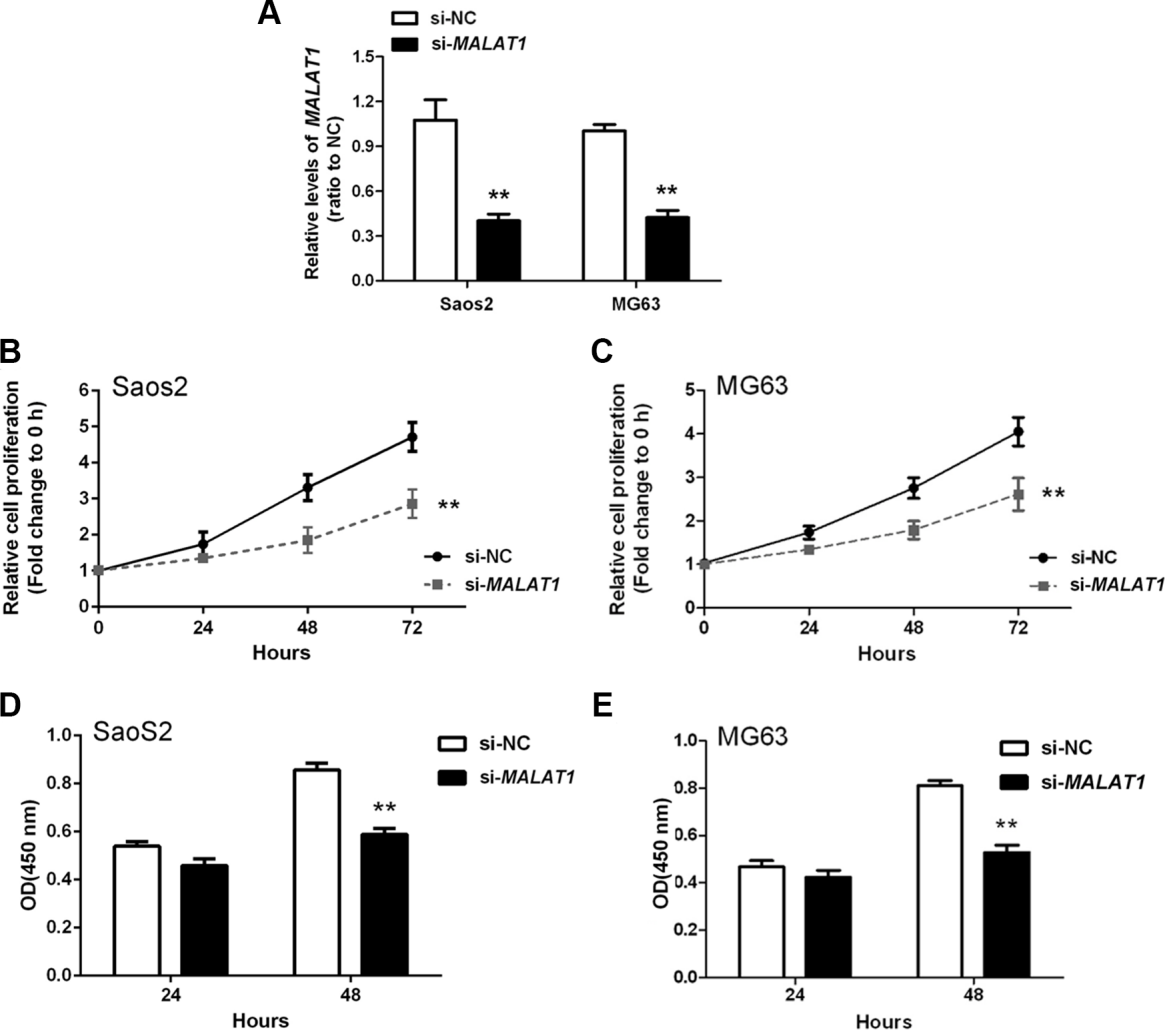
*MALAT1* promotion of osteosarcoma cell growth *in vitro* (**A**) *MALAT1* knockdown was achieved by si-*MALAT1* and the inhibitory efficiency was verified by real-time PCR. (**B** and **C**) MTT assays revealed that knockdown of *MALAT1* attenuated the growth of both Saos2 and MG63 cell lines up to three days, compared with si-NC group. (**D** and **E**) BrdU assays revealed that knockdown of *MALAT1* markedly reduced DNA synthesis in both Saos2 and MG63 cell lines. Data are presented as mean ± SD of three independent experiments.

To determine the association of *MALAT1* expression with OS cell proliferation and DNA synthesis, *MALAT1* siRNA (si-*MALAT1*), as well as negative control (si-NC), were transfected into two human OS cell lines: Saos2 and MG63. Compared with the si-NC group, *MALAT1* expression was decreased in cells transfected with si-*MALAT1* as measured by real-time qPCR (Figure 2A). Cell proliferation and DNA synthesis were determined by MTT and BrdU assays, respectively. When compared with the si-NC group, knockdown of *MALAT1* attenuated the growth of both Saos2 and MG63 cells up to three days (Figure 2B and 2C). Knockdown of *MALAT1* also reduced DNA synthesis in both Saos2 and MG63 cells (Figure 2D and 2E). Together, these data indicated that si-*MALAT1* successfully knocked-down *MALAT1* expression, and that *MALAT1* promotes OS cell growth and DNA synthesis.

### *MIR376A* suppression of osteosarcoma cell growth *in vitro*

**Figure 3:**
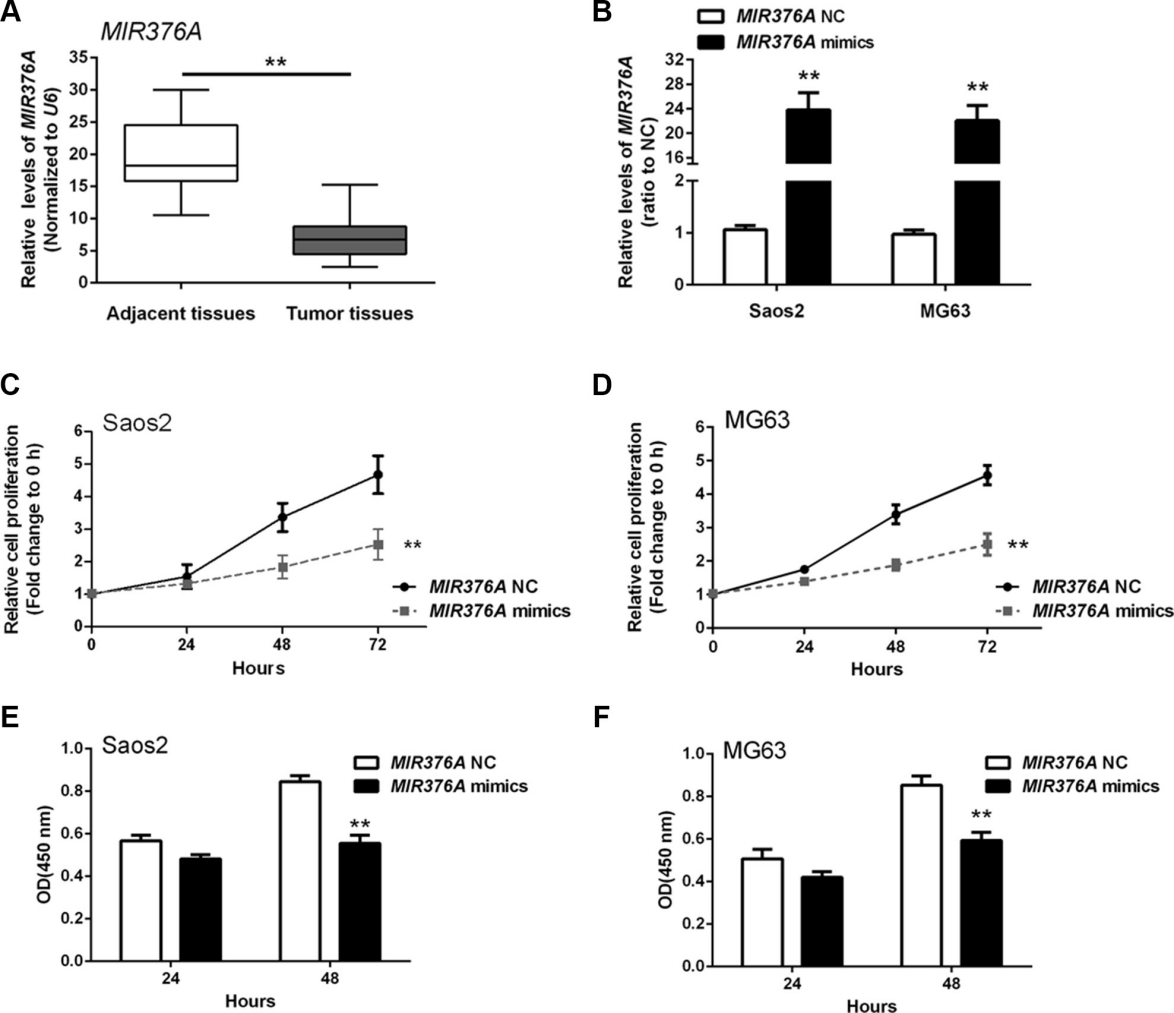
*MIR376A* suppression of osteosarcoma cell growth *in vitro* (**A**) Expression of *MIR376A* was downregulated in OS tissues compared with adjacent normal tissues. (**B**) *MIR376A* mimics were used to achieve *MIR376A* overexpression as confirmed by real-time qPCR in Saos2 and MG63 cell lines. (**C** and **D**) Cell growth of both Saos2 and MG63 cell lines were reduced in response to *MIR376A* overexpression compared with *MIR376A* NC group. (**E** and **F**) Results from BrdU incorporation assays indicated that *MIR376A* overexpression markedly reduced DNA synthesis in both Saos2 and MG63 cell lines compared with the *MIR376A*-NC group. Data are presented as mean ± SD of three independent experiments.

*MIR376A* was recently shown to function as a tumor suppressor in several cancers [16, 17]. To investigate the role of *MIR376A* in OS, we first examined *MIR376A* expression in OS tissues. *MIR376A* was downregulated in OS tissues compared with adjacent normal tissues (Figure 3A). *MIR376A* mimics were then transfected into Saos2 and MG63 cells to achieve *MIR376A* overexpression as confirmed by real-time qPCR (Figure 3B). MTT assays on the *MIR376A* overexpressing Saos2 and MG63 cells revealed that overexpression of *MIR376A* reduced cell growth when compared with the *MIR376A*-NC group (Figure 3C and 3D). In addition, *MIR 376A* overexpression reduced DNA synthesis in both Saos2 and MG63 cells compared with the *MIR376A*-NC group, as indicated by BrdU incorporation (Figure 3E and 3F). These data demonstrated that *MIR376A* inhibited OS cell growth and proliferation.

### Inverse correlation of *MIR376A* and *MALAT1* expression in osteosarcoma tissues and direct interaction between *MIR376A* and the 3′UTR of *MALAT1 in vitro*

**Figure 4:**
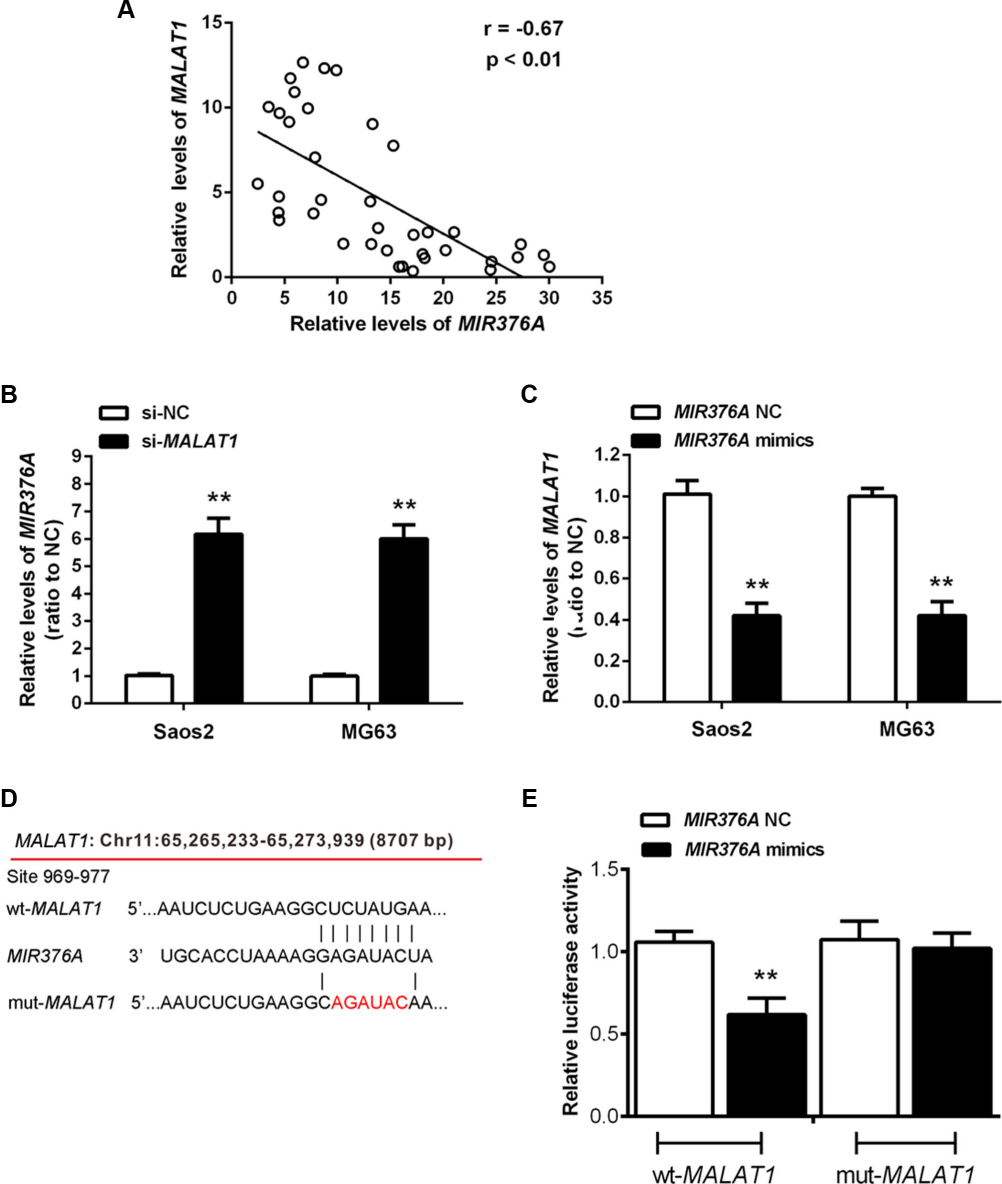
Inverse association of *MIR376A* with *MALAT1* expression in osteosarcoma tissues and direct interaction between *MIR376A* and the 3′UTR of *MALAT1 in vitro* (**A**) An inverse correlation between *MALAT1* and *MIR376A* expression was observed. (**B**) Real-time PCR assay showed that knockdown of *MALAT1* caused upregulation of *MIR376A*. (**C**) *MALAT1* expression was decreased in response to *MIR376A* overexpression, compared with the *MIR376A*-NC group. Data are presented as mean ± SD of three independent experiments. (**D**) Generation of wt-*MALAT1* and mut-*MALAT1* containing luciferase reporter vectors by sequentially mutating the predicted *MIR376A* binding site in the *MALAT1* 3′ untranslated region. (**E**) The wt-*MALAT1*/mut-*MALAT1* vectors and *MIR376A*-NC/*MIR376A* mimics were co-transfected into Saos2 cells, respectively. Luciferase activity of the wt-*MALAT1* vector was reduced in cells co-transfected with *MIR376A* mimics. Repression of luciferase activity by *MIR376A* was not shown in cells transfected with mut-*MALAT1*. Data are presented as mean ± SD of three independent experiments.

According to previous studies, *MIR376* family members play a suppressive role in OS [18, 19]. To investigate whether *MIR376A* correlates with *MALAT1* in regulation of OS cell growth, we performed expression analysis and found an inverse correlation between *MALAT1* and *MIR376A* expression in OS tissues (Figure 4A). Knockdown of *MALAT1* caused upregulation of *MIR376A* (Figure 4B), whereas *MIR376A* overexpression resulted in decreased *MALAT1* expression compared with the *MIR376A*-NC group (Figure 4C). Together, these data suggested that *MIR376A* expression is negatively correlated with *MALAT1* expression in OS.

To investigate whether *MIR376A* directly binds to *MALAT1*, we generated two luciferase reporter constructs: a wt-*MALAT1* and a mut-*MALAT1*. The mut-*MALAT1* contained a 6 bp mutation in the putative *MIR376A* binding site within the *MALAT1* 3′-UTR (Figure 4D). These wt-*MALAT1* and mut-*MALAT1* vectors and *MIR376A*-NC or *MIR376A* mimics were co-transfected into Saos2 cells. When compared with the control groups, luciferase activity of the wt-*MALAT1* vector was reduced in cells transfected with *MIR376A* mimics (Figure 4E). The repression of luciferase activity by *MIR376A* was not seen in cells transfected with mut-*MALAT1* (Figure 4E). These results suggested a direct interaction between *MIR376A* and *MALAT1* via the 6-bp putative *MIR376A* binding site within the 3′UTR of *MALAT1*.

### Upregulation of *TGFA* expression in osteosarcoma tissues and cells and its correlation with *MIR376A* and *MALAT1*

**Figure 5:**
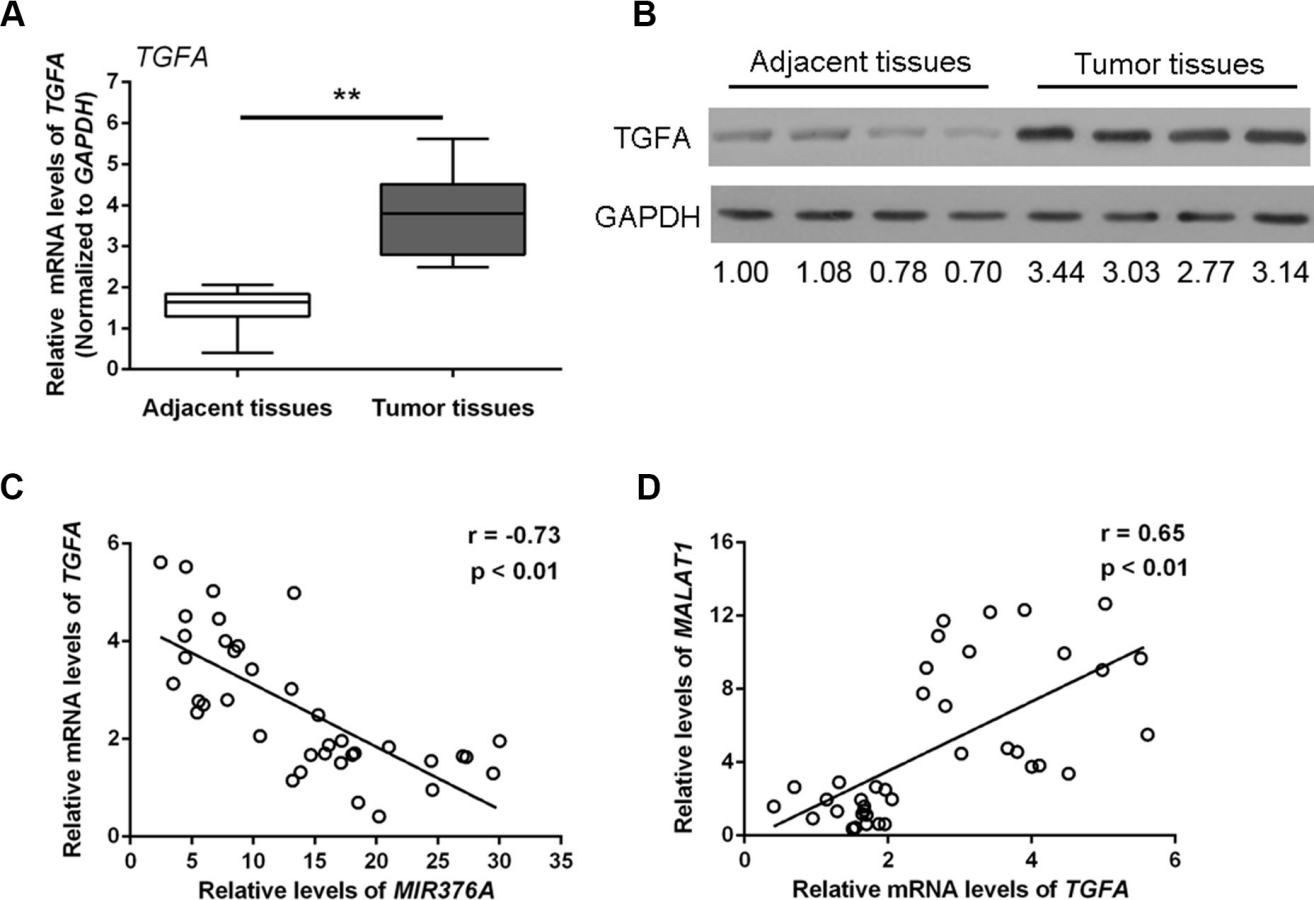
Upregulation of *TGFA* expression in osteosarcoma tissues and cells and its correlation with *MIR376A* and *MALAT1* (**A**) Expression of *TGFA* mRNA was upregulated in tumor tissues compared with adjacent normal tissues. (**B**) Expression of TGFA protein was upregulated in tumor tissues compared with adjacent normal tissues. (**C**) An inverse correlation between *TGFA* and *MIR376A* expression was observed. (**D**) A positive correlation between *MALAT1* and *TGFA* expression was observed. Data are presented as mean ± SD of three independent experiments.

*TGFA* is a direct target of *MIR376C*, one of the *MIR376* family members that regulate OS cell growth [18]. To investigate whether *TGFA* associates with *MIR376A* and *MALAT1* and plays a role in OS, we examined *TGFA* mRNA and protein expression in OS tissues. Both *TGFA* mRNA expression (Figure 5A) and TGFA protein expression (Figure 5B) were upregulated in tumor tissues compared with adjacent normal tissues. Thus, there was an inverse correlation between *TGFA* and *MIR376A* (Figure 5C), whereas *TGFA* expression was positively correlated with *MALAT1* expression (Figure 5D).

### *TGFA* promotion of osteosarcoma cell growth *in vitro*

**Figure 6:**
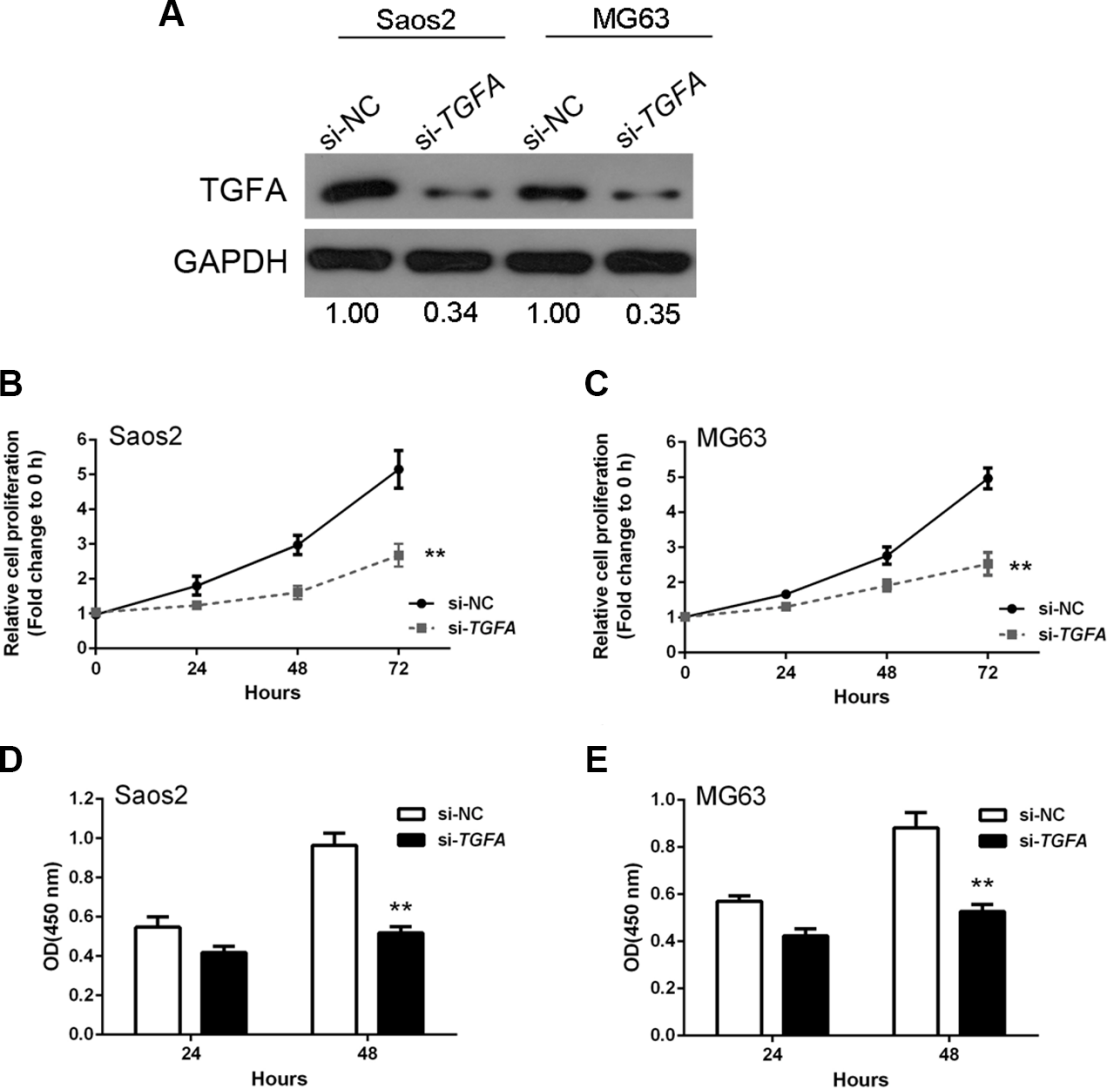
*TGFA* promotion of osteosarcoma cell growth *in vitro* (**A**) *TGFA* knockdown was achieved by si-*TGFA* as demonstrated by Western blot assay which showed much less protein expression of TGFA. (**B** and **C**) MTT assay results showed that OS cell growth was attenuated in response to *TGFA* inhibition by si-*TGFA*. (**D** and **E**) BrdU results showed that DNA synthesis capacities were reduced after *TGFA* inhibition. Data are presented as mean ± SD of three independent experiments.

We next investigated the effect of *TGFA* on OS cell growth by knockdown of *TGFA* in OS cells. *TGFA* was successfully knocked-down by si-*TGFA,* as demonstrated by western blots showing less TGFA protein expression (Figure 6A). MTT assays revealed that OS cell growth was attenuated in response to *TGFA* inhibition by si-*TGFA* (Figure 6B and 6C). DNA synthesis in both Saos2 and MG63 cells were also reduced after *TGFA* inhibition, as indicated by BrdU incorporation (Figure 6D and 6E). Together, these results suggested that *TGFA* promotes OS cell growth and proliferation.

### Regulation of *TGFA* by *MALAT1* and *MIR376A* in human OS cells and *TGFA* as a direct target of *MIR376A*

**Figure 7:**
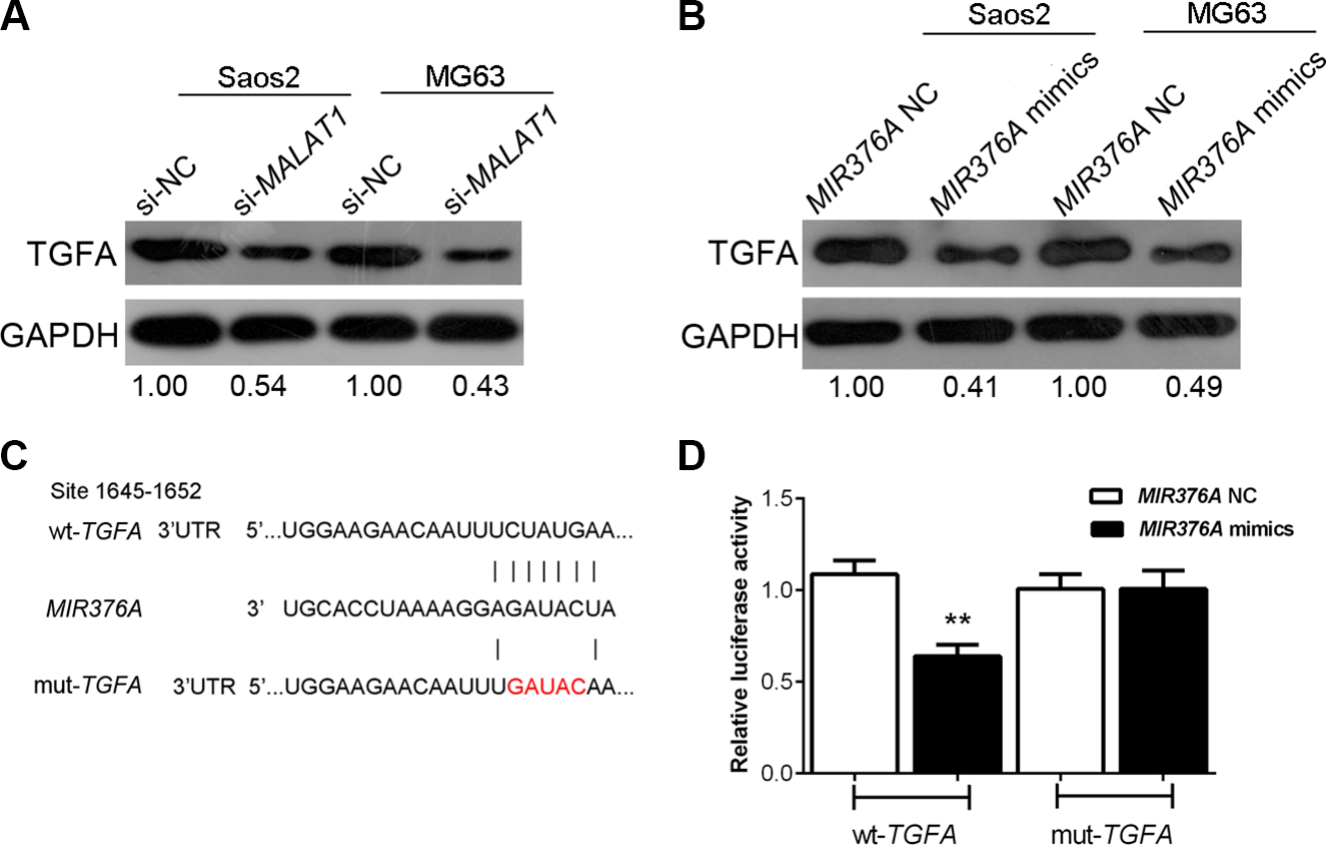
Regulation of *TGFA* by manipulation of *MALAT1* and *MIR376A* in human OS cells and *TGFA* as a direct target of *MIR376A* (**A**) Western blot assay showed that the expression of TGFA was downregulated by *MIR376A* overexpression in both Saos2 and MG63 cell lines. (**B**) Western blot results showed that knockdown of *MALAT1* also downregulated TGFA in both Saos2 and in MG63 cells. (**C**) Generation of wt-*TGFA* and a corresponding mut-*TGFA* containing a 5 bp mutation in a putative *MIR376A* binding site within its 3′-UTR. (**D**) The wt-*TGFA*/mut-*TGFA* vectors and *MIR376A*-NC/*MIR376A* mimics were co-transfected into Saos2 cells, respectively. The luciferase activity of the wt-*TGFA* reporter was reduced by co-transfection with *MIR376A* mimics, as compared with the control groups. No reduction of reporter activity was shown in cells co-transfected with *MIR376A* and the mut-*TGFA* reporter. Data are presented as mean ± SD of three independent experiments.

*TGFA* expression was negatively correlated with *MIR376A* but positively correlated with *MALAT1* expression (Figure 5B and 5C). To further determine their relationship, we measured the expression of *TGFA* in response to *MIR376A* overexpression and *MALAT1* knockdown in human OS cells. TGFA was downregulated by overexpression of *MIR376A* as demonstrated by western blot (Figure 7A). Knockdown of *MALAT1* also downregulated TGFA in Saos2 and MG63 cells (Figure 7B).

To investigate whether *MIR376A* repression of OS cell growth is through inhibition of *TGFA*, we generated two *TGFA*-containing luciferase reporter constructs: wt-*TGFA* and a mut-*TGFA* which contained a 5 bp mutation in a putative *MIR376A* binding site within its 3′-UTR (Figure 7C). These luciferase reporter constructs were co-transfected into Saos2 cells with *MIR376A*-NC or *MIR376A* mimics. The luciferase activity of the wt-*TGFA* reporter was reduced by transfection of *MIR376A* mimics when compared with the control groups (Figure 7D). There was no reduction of reporter activity in cells co-transfected with *MIR376A* and the mut-*TGFA* reporter, suggesting that *MIR376A* directly inhibits *TGFA* through interaction with its 3′-UTR (Figure 7D). These results support a direct correlation of *MALAT1*, *MIR376A*, and *TGFA* expression in the regulation of OS cell growth.

## DISCUSSION

The lncRNA *MALAT1* is upregulated in many cancers, including OS [12, 13, 20]. *MALAT1* has been shown to promote cancer cell proliferation in a variety of malignancies and acts as an oncogene in renal cancer [21]. *MALAT1* promotes tumor-driven angiogenesis by upregulating pro-angiogenic gene expression in neuroblastoma [22]. In addition, *MALAT1* may also promote colorectal cancer development by directly targeting *AKAP9* [23]. In our study, we showed that *MALAT1* expression was higher in all four OS cell lines and tissues compared with normal cell lines and adjacent normal tissues. We also found that knockdown of *MALAT1* by siRNA resulted in decreased cell proliferation and DNA synthesis in OS cells. These results suggested that *MALAT1* functions as an oncogene in OS by promoting OS cell growth, and that *MALAT1* might be related with tumor progression.

*MIR376A* is a tumor-suppressive microRNA associated with multiple cancers, including hepatocellular carcinoma, melanoma, and glioblastoma [16, 17, 24, 25]. *MIR376A* maps to the 14q32 locus, which harbors a cluster of miRNAs that are known to regulate proliferation, apoptosis, migration, and invasion of several cancers [24, 26]. We found that *MIR376A* expression was downregulated in OS tissues, and *MIR376A* overexpression inhibited OS cell growth and proliferation. We also observed an inverse correlation between *MALAT1* and *MIR376A* expression in OS tissues. Knock-down of *MALAT1* upregulated *MIR376A*, while overexpression of *MIR376A* inhibited *MALAT1* expression *in vitro*, suggesting a direct association between *MALAT1* and *MIR376A*. In support of this, we found that *MIR376A* may regulate *MALAT1* via a putative binding site within its 3′UTR. These results provide direct evidence of a *MALAT1*-*MIR376A* interaction in the regulation of OS tumorigenesis. Interestingly, it has been shown that *MALAT1* promotes proliferation and metastasis in kidney renal cell carcinoma, possibly through inhibition of *MIR200S*, as forced overexpression of *MIR200S* partially attenuated the effects of *MALAT1* on growth and metastasis [27]. Moreover, a negative correlation between *MIR101* or *MIR217* and *MALAT1* was previously observed in esophageal squamous cell carcinoma, while knockdown of *MALAT1* inhibited cell growth, migration, and invasion [28]. These studies, along with our data, suggest that a lncRNA-miRNA interaction might be important in the process of tumorigenesis.

It has been reported that *TGFA* regulates OS cell growth as a direct target of *MIR376C*, one of the *MIR376* family members [18]. Our data indicated that *TGFA* expression was increased in OS tissues compared with adjacent non-tumor tissues and that *TGFA* promoted OS cell growth *in vitro*. We also showed that *TGFA* expression is positively correlated with *MALAT1* but negatively correlated with *MIR376A*. Moreover, overexpression of *MIR376A* or knockdown of *MALAT1* both resulted in markedly reduced TGFA expression. There was a direct interaction between *MIR376A* and *TGFA*, with *MIR376A* regulating *TGFA* via a 5-bp putative binding site within its 3′-UTR. Previous studies have shown that *TGFA* promotes OS cell growth, invasion and migration, and support our findings [18, 29]. Recently, other studies have indicated that the interaction between *TGFA* and miRNAs play an essential role in OS tumorigenesis [18, 29]. Similar to our findings, few studies have also shown that lncRNA-miRNA interactions, for example, the *H19*-*MIR675* interaction and the *MALAT1*-MIR9 interaction, are important singling pathways in the process of OS tumorigenesis [30, 31]. Thus, our data indicate that *MALAT1* may promote OS cell growth through inhibition of *MIR376A* and by targeting *TGFA*. This is the first time that lncRNAs, miRNAs, and *TGFA* have been linked in OS *in vitro*, which warrants further studies to verify this finding in animal models.

In conclusion, we found differential expression of *MALAT1*, *MIR376A* and *TGFA* in OS cell lines and tissues. All three genes have been associated with OS tumor progression. We showed that *TGFA* expression correlated with *MALAT1* and *MIR376A* expression in OS. More importantly, there was a direct interaction between *MIR376A* and *MALAT1* or *TGFA*. Our results support a *MALAT1*/*MIR376A*/*TGFA* axis in OS tumor progression whereby *MALAT1* promotes OS cell growth through inhibition of *MIR376A* and targeting of *TGFA*.

## MATERIALS AND METHODS

### Cell lines

Human OS cell lines, Saos2, MG63, U2OS, SW1353, and normal cells, hFOB, were purchased from American Type Culture Collection.

### Tissue specimens

Thirty-eight paired OS specimens and corresponding adjacent non-tumor tissues were collected from tumor surgical resection in Xiangya Hospital of Central South University (Changsha, China). All the human tissues were obtained with informed consent and this study was approved by the Clinical Research Ethics Committee of Xiangya Hospital of Central South University.

### Cell transfection

Cells were seeded in 6-well plates at a concentration of 2 × 10^5^ cells/well. When cells reached 40–60% confluence, 150-nM *MIR376A* mimics or negative control (NC) was transfected using Lipofectamine™ 2000 transfection reagent (Invitrogen, USA) following the protocol recommended by the manufacturer. The miRNA mimic and NC were synthesized by Shanghai GenePharma Co. (Shanghai, China). Their sequences were as follows: 5′-UUCUCCGAAC GUGUCACGUT T-3′ (sense) and 5′-ACGUGACACG UUCGGAGAAT T-3′ (antisense) for NC and 5′-AUCAUAGAGG AAAAUCCACG U-3′ (sense) and 5′-GUGGAUUUUC CUCUAUGAUU U-3′ (antisense) for *MIR376A* mimics. After 48 h transfection, the cells were collected and used for further analyses.

### MTT assay

Cell proliferation assay using the MTT kit (Promega Corporation, Madison, WI, USA) was performed according to the manufacturer's instructions. Briefly, cells were seeded into 96-well plates at a density of 5000 cells per well and grown for 24 hours. The cells were then transfected with 100 nM *MIR376A* mimics, *MIR376A*-NC, si-NC/si-*MALAT1* or si-NC/si-*TGFA*. After 24 h transfection, 20 μL of 5 mg/mL MTT was added and further incubated for 4 h in a humidified incubator. 200 μL of DMSO was added to dissolve the formazan after supernatant removed. Optical density (OD) was measured at 490 nm.

### BrdU incorporation assay

BrdU assays were performed to determine DNA synthesis at 24 h and 48 h after transfection of Saos2 and MG63 cells with designated constructs, miRNAs, or siRNAs. After transfection, cells were incubated with a final concentration of 10 μM BrdU (BD Pharmingen, San Diego, CA, USA) for 2 to 24 h, followed by fixation for 30 min after removing the medium. Cells were then incubated with peroxidase-coupled anti-BrdU-antibody (Sigma-Aldrich) for 60 min, washed with PBS and further incubated with peroxidase substrate (tetramethylbenzidine) for 30 min. Absorbance values were measured at 450 nm.

### Western blot

RIPA buffer (Sigma-Aldrich, USA) was used to lyse cells with Complete Protease Inhibitor Cocktail (Roche, USA). Cell lysates were transferred to 1.5 mL tube and kept at −20°C before use. SDS-PAGE was conducted to separate the cellular proteins. Proteins were separated by 5% stacking gel and 10% running gel. The molecular weight of candidate proteins was referred to the Pre-stained SeeBlue rainbow marker (Invitrogen, USA) loaded in parallel. The following antibodies were used: *MALAT1* (Santa Cruz, USA), TGFA (Abcam, MA, USA), and β-actin (Sigma, USA). Blots were detected using a Kodak film developer (Fujifilm, Japan).

### RNA extraction and real-time PCR

Total RNA was extracted using TRIZOL reagent (Invitrogen, USA) following the manufacturer's instructions. A High Capacity cDNA Reverse Transcription Kit (Applied Biosystems, USA) was used to reversely transcribe RNA samples. Quantitative RT-PCR was performed using the Fast Start Universal SYBR Green Mastermix (Roche, USA). Primers are shown in Table 1. The relative fold changes of candidate genes were analyzed using the 2^−ΔΔ^CT method.

**Table 1:** The primers used for real-time PCR

Name		Sequences
miR-376a	Forward	5′- GTGCAGGGTCCGAGGT-3′
	Reverse	5′- ATCATAGAGGAAAATCCACG -3′
MALAT1	Forward	5′-AAAGCAAGGTCTCCCCACAAG-3′
	Reverse	5′-GGTCTGTGCTAGATCAAAAGGCA-3′
TGF-α	Forward	5′-AGCTGCTAGCGCCTAGCGAT-3′
	Reverse	5′-CCCGTCTGATAGCGCATTCGTGT-3′
GAPDH	Forward	5′-AGAAGGCTGG GGCTCATTTG-3′
	Reverse	5′-AGGGGCCATC CACAGTCTTC-3′

### Luciferase reporter assays

The 3′-UTR or mutant 3′-UTR of *MALAT1* containing the putative target site for *MIR376A* was chemically synthesized and inserted downstream of the luciferase gene in the internal control pRSV-β-Galactosidase vector. Saos2 cells cultured in 24-well plates were co-transfected with luciferase reporter plasmids and miRNA mimics as well as the internal control pRSV-β-Galactosidase vector. After transfection for 48 h, Saos2 cells were lysed with lysis buffer (25 mM Tris-phosphate, 1% Triton X-100, 1 mM DTT, 2 mM EDTA, 10% Glycerol, pH = 27.8). Cells were then collected and centrifuged at 14,000 rpm for 3 min, and the supernatant transferred to a new 1.5 μL tube. Luciferase reporter activity was monitored by mixing 50μL supernatant with 50 μL luciferase assay buffer using the Gloxmax 20/20 Luminometer (Promega). O-nitrophenyl-β-galactoside (ONPG) colorimetric assays were performed to measure the β-Galactosidase activity from the pRSV-β-Galactosidase vector, which was used for normalization of the luminescence levels. 50 μL supernatant from aforementioned cell extract was mixed with 100 μL of ONPG solution (0.666 mg/ml ONPG, 40 mM NaH2PO4, 60 mM Na2HPO4, 10 mM KCl, 1 mM MgSO_4_, 2% β-mercaptoethanol) and β-Galactosidase activity was measured using the ELISA plate reader (Bio-Rad, USA) at the wavelength of 490nm.

### Statistical analysis

Experimental results are presented as mean ± SD. Comparisons between two groups were conducted using two-tailed Student's *T*-test and differences were considered to be statistically significant when the *P* value was less than 0.05.
